# Hexaxial external fixator versus intramedullary nail in treating segmental tibial fractures: a retrospective study

**DOI:** 10.1186/s12893-024-02327-0

**Published:** 2024-02-01

**Authors:** Zhiming Zhao, Hengsheng Shu, Peng Jia, Xu Chen, Feng Guo, Yabin Liu, Bowen Shi, Guoqi Ji, Chengkuo Cai, Yidong Shen

**Affiliations:** 1 Department of Traumatic Orthopaedics, Tianjin Hospital, No 406, South Jiefang Road, Hexi District, Tianjin, 300211 China; 2 Department of Orthopaedics, The First People’s Hospital of Yancheng (Yancheng First Hospital, Affiliated Hospital of Nanjing University Medical School), Yancheng, China

**Keywords:** Tibial fractures, External fixators, Fracture fixation, Intramedullary

## Abstract

**Background:**

It’s difficult to treat segmental tibial fractures (STFs), which are intricate injuries associated with significant soft tissue damage. The aim of this study was to compare the clinical effect of hexaxial external fixator (HEF) and intramedullary nail (IMN) in treatment of STFs.

**Methods:**

A total of 42 patients with STFs were finally recruited between January 2018 and June 2022. There were 25 males and 17 females with age range of 20 to 60 years. All fractures were classified as type 42C2 using the Arbeitsgemeinschaftfür Osteosythese/Orthopaedic Trauma Association (AO/OTA) classification. 22 patients were treated with HEF and 20 patients were treated with IMN. The condition of vascular and neural injuries, time of full weight bearing, bone union time and infection rate were documented and analyzed between the two groups. The mechanical medial proximal tibial angle (mMPTA), mechanical posterior proximal tibial angle (mPPTA), mechanical lateral distal tibial angle (mLDTA), mechanical anterior distal tibial angle (mADTA), hospital for special surgery (HSS) knee joint score, American Orthopaedic Foot and Ankle Society (AOFAS) ankle joint score, range of motion (ROM) of flexion of keen joint and ROM of plantar flexion and dorsal flexion of ankle joint were compared between the two groups at the last clinical visit.

**Results:**

There were no vascular and neural injuries or other severe complications in both groups. All 22 patients in HEF group underwent closed reduction but 3 patients in IMN group were treated by open reduction. The time of full weight bearing was (11.3 ± 3.2) days in HEF group and (67.8 ± 5.8) days in IMN group(*P* < 0.05), with bone union time for (6.9 ± 0.8) months and (7.7 ± 1.4) months, respectively(*P* < 0.05). There was no deep infection in both groups. In the HEF group and IMN group, mMPTA was (86.9 ± 1.5)° and (89.7 ± 1.8)°(*P* < 0.05), mPPTA was (80.8 ± 1.9)° and (78.6 ± 2.0)°(*P* < 0.05), mLDTA was (88.5 ± 1.7)° and (90.3 ± 1.7)°(*P* < 0.05), while mADTA was (80.8 ± 1.5)° and (78.4 ± 1.3)°(*P* < 0.05). No significant differences were found between the two groups at the last clinical visit concerning HSS knee joint score and AOFAS ankle joint score, ROM of flexion of keen joint and ROM of plantar flexion of ankle joint (*P* > 0.05). The ROM of dorsal flexion of ankle joint in IMN group was (30.4 ± 3.5)°, better than (21.6 ± 2.8)° in HEF group (*P* < 0.05).

**Conclusion:**

In terms of final clinical outcomes, the use of either HEF or IMN for STFs can achieve good therapeutic effects. While HEF is superior to IMN in terms of completely closed reduction, early full weight bearing, early bone union and alignment. Nevertheless, HEF has a greater impact on the ROM of dorsal flexion of the ankle joint, and much more care and adjustment are needed for the patients than IMN.

## Background

STFs constitute approximately 12% of tibial shaft fractures. These fractures are intricate, high-energy injuries distinguished by the presence of distinct fractures at two or more levels, creating a separate intercalary diaphyseal fragment of bone [1]. Typically, they are associated with wide zones of soft tissue damage [2]. Surgical treatments include plate osteosynthesis, IMN, and external fixation [3–5]. IMN is a commonly used method of fixation for STFs, but there are drawbacks, such as high operational difficulty, high reduction technical requirements, and poor stability [4]. External fixation can provide stability for multi-plane fracture fixation and minimize local soft tissue damage. Traditional Ilizarov circular external fixation has advantages such as reliable fixation, minimal trauma, and precise therapeutic effects and is especially suitable for open fractures and STFs with severe soft tissue damage [6, 7]. However, the circular external fixator has disadvantages such as a complex operation and a long learning curve [8]. HEF is developed based on the Ilizarov external fixation system, which is an external fixation system that places 6 universal adjustment rods between two rings. The spatial configuration can be changed by adjusting the length of the 6 universal adjustment rods, thereby achieving reduction and correction of the fracture site [9, 10]. In recent years, the application of HEF in fracture reduction, limb correction, and functional reconstruction of patients in China has gradually increased [11–13]. However, there is currently a lack of comparative research on the efficacy of HEF and IMN in the treatment of STFs. Consequently, the aim of this study was to compare the clinical effect of HEF and IMN in the treatment of STFs.

## Methods

### Patients

We evaluated all consecutive patients with STFs according to the inclusion and exclusion criteria from January 2018 to June 2022 at our department of traumatic Orthopaedics.

The inclusion criteria were as follows: (1) the AO/OTA classification of the tibia was type 42C2, (2) age range of 22 to 60 years old, and (3) patients treated with HEF or IMN. The exclusion criteria were as follows: (1) the fracture line affected the proximal and distal articular surfaces of the tibia, (2) patients with diabetes, cardiovascular disease and other medical diseases that seriously affected the surgical treatment effect and prognosis, (3) patients showed poor compliance and failed to wear external fixation, (4) patients with fracture(s) in other limbs, and (5) patients with incomplete follow-up and follow-up time less than 12 months.

A total of 42 patients with STFs were finally evaluated. There were 25 males and 17 females with an age range of 20 to 60 years. All fractures were classified as type 42C2 using the AO/OTA classification. 5 patients had skin and soft tissue defects with a defect area of 3 cm × 3 cm to 3 cm × 6 cm. All patients were accompanied by varying degrees of fibular fractures. 3 patients were combined with ipsilateral posterior ankle fractures. 22 patients were treated with HEF, and 20 patients were treated with IMN. There was no statistically significant difference in demographics between the two groups of patients (*P* > 0.05), indicating comparability (Table 1). The study protocol was approved by the ethics committee of Tianjin Hospital. The study was conducted in accordance with the principles of the Declaration of Helsinki.

**Table 1:** Demographics in the two groups

Variable	HEF(*n* = 22)	IMN(*n* = 20)	*P-*value
Gender			0.569
Male	14(63.6)	11(55.0)	
Female	8(36.4)	9(45.0)	
Age (year)	46.00 ± 9.7	41.80 ± 12.1	0.220
Injury mechanism			0.435
Traffic accident injury	15(68.2)	10(50.0)	
High falling injury	2(9.1)	4(20.0)	
Crushing injury	5(22.7)	6(30.0)	
Soft tissue damage			0.973
Skin integrity	9(40.9)	8(40.0)	
Blunt contusion	7(31.8)	7(35.0)	
Open injury	6(27.3)	5(25.0)	
Open/closed fracture			0.867
Open	6(27.3)	5(25.0)	
Closed	16(72.7)	15(75.0)	
Gustilo-Anderson grading			0.885
Type I	3(13.6)	3(15.0)	
Type II	2(9.1)	1(5.0)	
Type III	1(4.5)	1(5.0)	
Time elapsed since the injury to definitive treatment (day)	5.32 ± 2.7	6.00 ± 2.7	0.419

Data are expressed as the mean ± SD or frequency (%); *HEF* Hexaxial external fixator, *IMN* Intramedullary nail

### Surgical technique

Under general or epidural anesthesia, the patients were placed in a supine position on the radiolucent operating table and received antibiotic prophylaxis.

HEF group: HEF (Shanghai Carefix Medical Instrument Co., Ltd., Shanghai, China) was used, and the number and fixation position of metal rings were determined based on different positions of the fracture line. Each bone segment was fixed with wires separately, and each metal ring was placed as perpendicular to the bone segment as possible. Each ring of the tibia was crossed with 2–3 2.0-mm olive wires, tensioned and fixed on the ring, and each ring was strengthened with 1–2 6-mm hydroxyapatite (HA)-coated half wires. Six quick universal adjustment rods were used to connect every two sets of rings and placed in a sliding state. The fracture was manually closed for reduction, and the quick universal adjustment rods were then locked. The residual fracture displacement could be adjusted by measuring the anteroposterior (AP) and lateral radiographs with the aid of computer-based software postoperatively.

IMN group: Point-shaped reduction forceps were used to reduce the fracture. Split the patellar tendon longitudinally with a subpatellar approach, drilled a hole at the slope in front of the tibial plateau, inserted a guide wire, sequentially expanded the medullary cavity, selected an IMN of appropriate length and diameter (Smith & Nephew, USA), and inserted it into the medullary cavity. If the medullary cavity was wide, blocking screw technology was used, or the skin and subcutaneous tissue were opened for reduction of the fracture when necessary. After installing the sight, locking screws were placed in the far and near ends, the tail cap was screwed in, and the incision was cleaned and sutured.

In the emergency stage of open fractures, wound debridement, suture, and calcaneal traction were performed. After the wound showed no inflammatory symptoms, such as redness, swelling, heat, and pain, and the white blood cells, erythrocyte sedimentation rate, and C-reactive protein reached or nearly reached normal, the second-stage IMN internal fixation surgery was performed 1–4 days after debridement.

For patients whose wounds could not be completely sutured and closed, local flap transfer on the lower leg was performed to cover the wound, or vacuum sealing drainage (VSD) was applied, followed by skin grafting or dressing change. Fibular fractures needed neither internal nor external fixation. For patients with combined posterior ankle fractures, hollow screws (Stryker, USA) were inserted from front to back after reduction for fixation.

### Postoperative management

HEF group: Pin tract care was performed by dripping 75% ethanol daily once a day to prevent infection. If the pin tract was dry and did not exude, it could be stopped. On the first day after the operation, isometric contraction training of the quadriceps femoris muscle and knee and ankle flexion and extension activities were started. On the second day after the operation, patients were encouraged to get out of bed and exercise. With the help of double crutches, they were able to stand and then gradually walk with weight bearing. The residual deformities could be gradually adjusted by measuring the AP and lateral radiographs with the aid of computer-based software postoperatively within 3 days.

IMN group: On the first day after the operation, each patient underwent active and passive flexion and extension exercises for the knee and ankle joints. Two weeks after the operation, the stitches were removed. According to the X-ray showing the formation of a callus, with the help of double crutches, the patients were able to stand and then gradually walk with weight bearing after 2–3 weeks of operation, and complete weight bearing was allowed after the fracture sites healed.

A monthly follow-up and radiograph were conducted for both groups.

### Evaluation

The condition of vascular and neural injuries, time of full weight bearing, bone union time and infection rate were documented and analyzed between the two groups. The mMPTA, mPPTA, mLDTA, mADTA, HSS knee joint score, AOFAS ankle joint score, ROM of flexion of the knee joint and ROM of plantar flexion and dorsal flexion of the ankle joint were compared between the two groups at the last clinical visits. The HSS knee joint score includes pain, motor function, ROM of the joint, muscle strength, flexion deformity, and stability, with a total score of 100 points. A total of 85–100 points are excellent, 70–84 points are good, 60–69 points are acceptable, and below 59 points are poor; the AOFAS ankle joint score includes pain, function and self-activity, support situation, maximum walking distance (blocks), ground walking, abnormal gait, etc., with a total score of 100 points. A total of 90–100 points are excellent, 75–89 points are good, 50–74 points are acceptable, and below 50 points are poor.

### Statistical analysis

Data analysis was performed by using SPSS 21.0 statistical software (IBM Corp, USA). Continuous variables were retrieved and analyzed. Data were presented as the mean ± SD (standard deviation) or frequency (%). The difference between the two groups was analyzed by Student *t*-test or Chi-square test where appropriate, and the difference was statistically significant at *P* < 0.05.

## Results

All patients were followed up for 12–18 months. There were no vascular or neural injuries or other severe complications in either group. All 22 patients in the HEF group underwent closed reduction, but 3 patients in the IMN group were treated by open reduction. The time of full weight bearing was 11.3 ± 3.2 days in the HEF group and 67.8 ± 5.8 days in the IMN group (*P* < 0.05), with bone union times of 6.9 ± 0.8 months and 7.7 ± 1.4 months, respectively (*P* < 0.05) (Table 2). There was no deep infection in either group. One patient in the IMN group showed no obvious signs of healing at the fracture site 8 months after the operation. The fracture site was cleaned, and iliac bone grafting was performed to assist in internal fixation with a steel plate and screw (WEGO, CHINA). The fracture finally healed 5 months after the operation. In the HEF group, 7 patients experienced superficial wire infection after the operation. The infection was controlled after pin-site dressing and oral antibiotics. 2 patients in the IMN group with open fractures experienced postoperative redness and swelling around the wound. After dressing changes and the application of antibiotics, the wound healed smoothly, and no deep wound infection occurred.

**Table 2:** Clinical outcomes in the two groups

Variable	HEF(*n* = 22)	IMN(*n* = 20)	*P-*value
Time of full weight bearing(d)	11.3 ± 3.2	67.8 ± 5.8	0.000
Bone union time(month)	6.9 ± 0.8	7.7 ± 1.4	0.000
mMPTA(°)	86.9 ± 1.5	89.7 ± 1.8	0.016
mPPTA(°)	80.8 ± 1.9	78.6 ± 2.0	0.011
mLDTA(°)	88.5 ± 1.7	90.3 ± 1.7	0.011
mADTA(°)	80.8 ± 1.5	78.4 ± 1.3	0.000

Data are expressed as the mean ± SD; *mMPTA* Mechanical medial proximal tibial angle, *mPPTA* Mechanical posterior proximal tibial angle, *mLDTA* Mechanical lateral distal tibial angle, *mADTA* Mechanical anterior distal tibial angle

At the last visit, both the mMPTA and mLDTA in the HEF group were within the normal range and lower than those in the IMN group (*P* < 0.05) (Table 2). The mPPTA and mADTA in the HEF group were both within the normal range and higher than those in the IMN group (*P* < 0.05) (Table 2). 9 patients in the HEF group had ankle joint partial mobility limitations when the external fixation was first removed. After removal of the external fixation, they took active rehabilitation exercise and showed significant improvement in ankle joint mobility at the last clinical visits. No significant differences were found between the two groups at the last clinical visits concerning the HSS knee joint score and AOFAS ankle joint score, ROM of flexion of the knee joint and ROM of plantar flexion of the ankle joint (*P* > 0.05). The ROM of dorsal flexion of the ankle joint in the IMN group was 30.4 ± 3.5°, which was better than the 21.6 ± 2.8° in the HEF group (*P* < 0.05) (Table 3).

**Table 3:** Clinical outcomes in the two groups

Variable	HEF(*n* = 22)	IMN(*n* = 20)	*P-*value
HSS knee joint score	90.8 ± 2.5	91.8 ± 2.5	0.206
AOFAS ankle joint score	87.2 ± 4.1	88.1 ± 2.5	0.410
ROM of flexion of keen joint (°)	117.1 ± 5.5	116.9 ± 5.6	0.912
ROM of plantar flexion of ankle joint (°)	31.3 ± 2.9	32.4 ± 3.8	0.283
ROM of dorsal flexion of ankle joint (°)	21.6 ± 2.8	30.4 ± 3.5	0.000

Data are expressed as the mean ± SD, *HSS* Hospital for Special Surgery, *AOFAS* American Orthopaedic Foot & Ankle Society

**Fig. 1:**
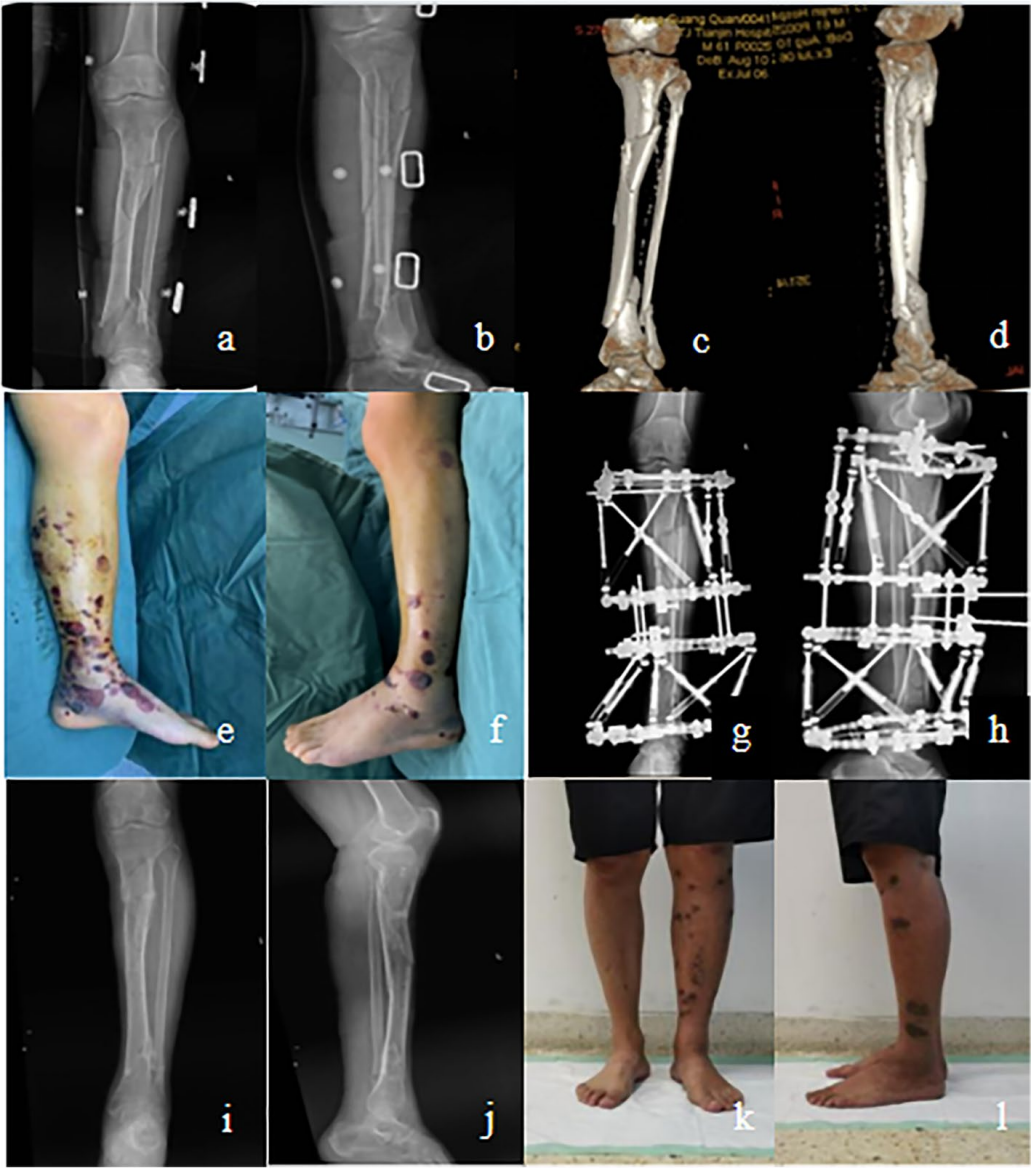
Images of treating a 38-year-old male with STFs due to high falling by using HEF. **a**, **b** X-ray images of injured STFs preoperatively. **c**, **d** Computer tomography images of injured STFs preoperatively. **e**, **f** Appearance images show poor local soft tissue conditions in the left lower leg preoperatively. **g**, **h** X-ray images within one week postoperatively. **i**, **j** X-ray images show good fracture healing, one week after removal of HEF. **k**, **l** Clinical follow-up appearance images obtained 1 month after HEF removal

**Fig. 2:**
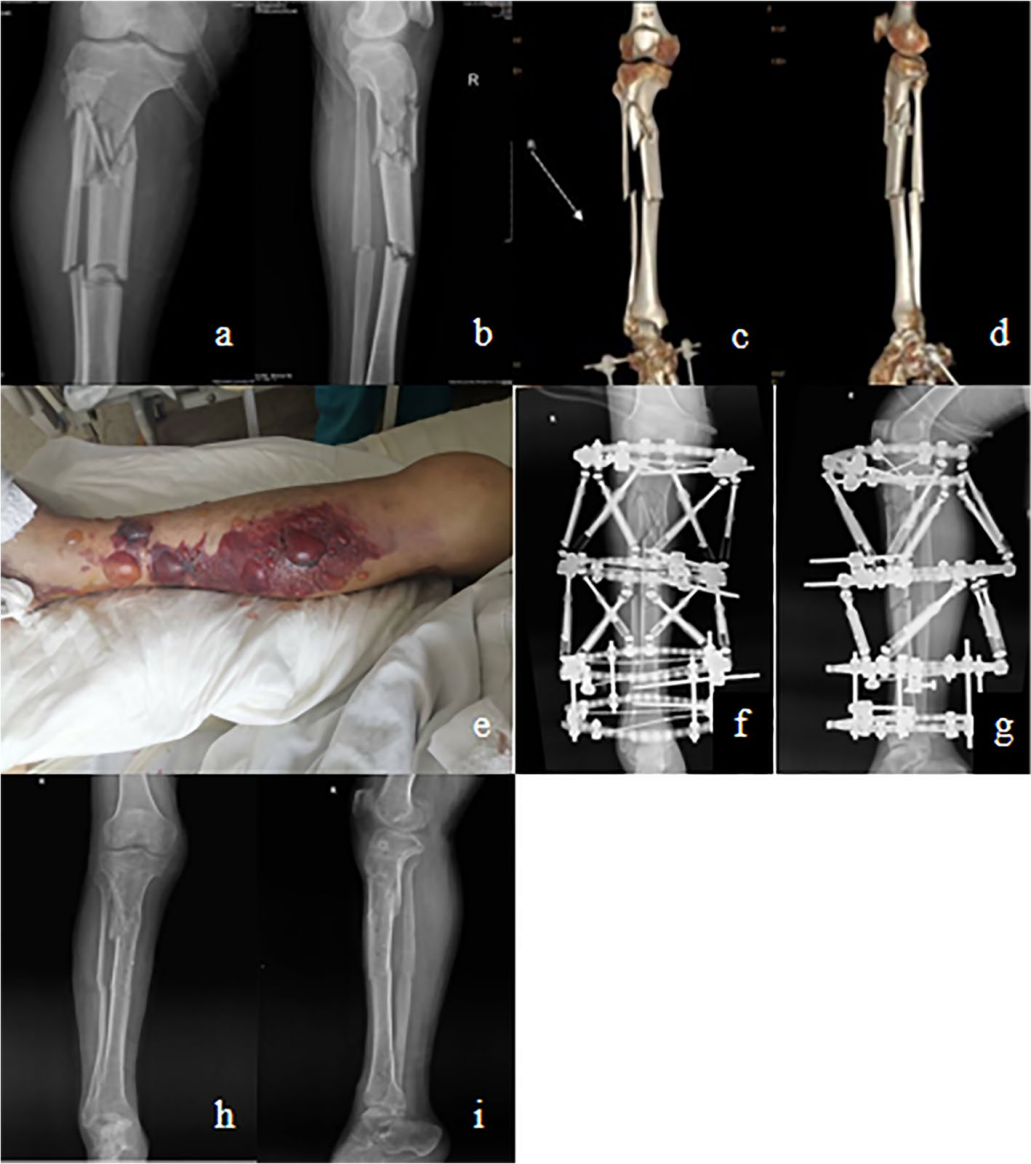
Images of treating a 45-year-old male with STFs due to traffic accident by using HEF. **a**, **b** X-ray images of injured STFs preoperatively. **c**, **d** Computer tomography images of injured STFs preoperatively. **e** Appearance image shows poor local soft tissue conditions in the right lower leg preoperatively. **f**, **g** X-ray images within one week postoperatively. **h**, **i** X-ray images show good fracture healing, one week after removal of HEF

Two typical STFs in HEF treatment are shown in Figs. 1 and 2.

**Fig. 3:**
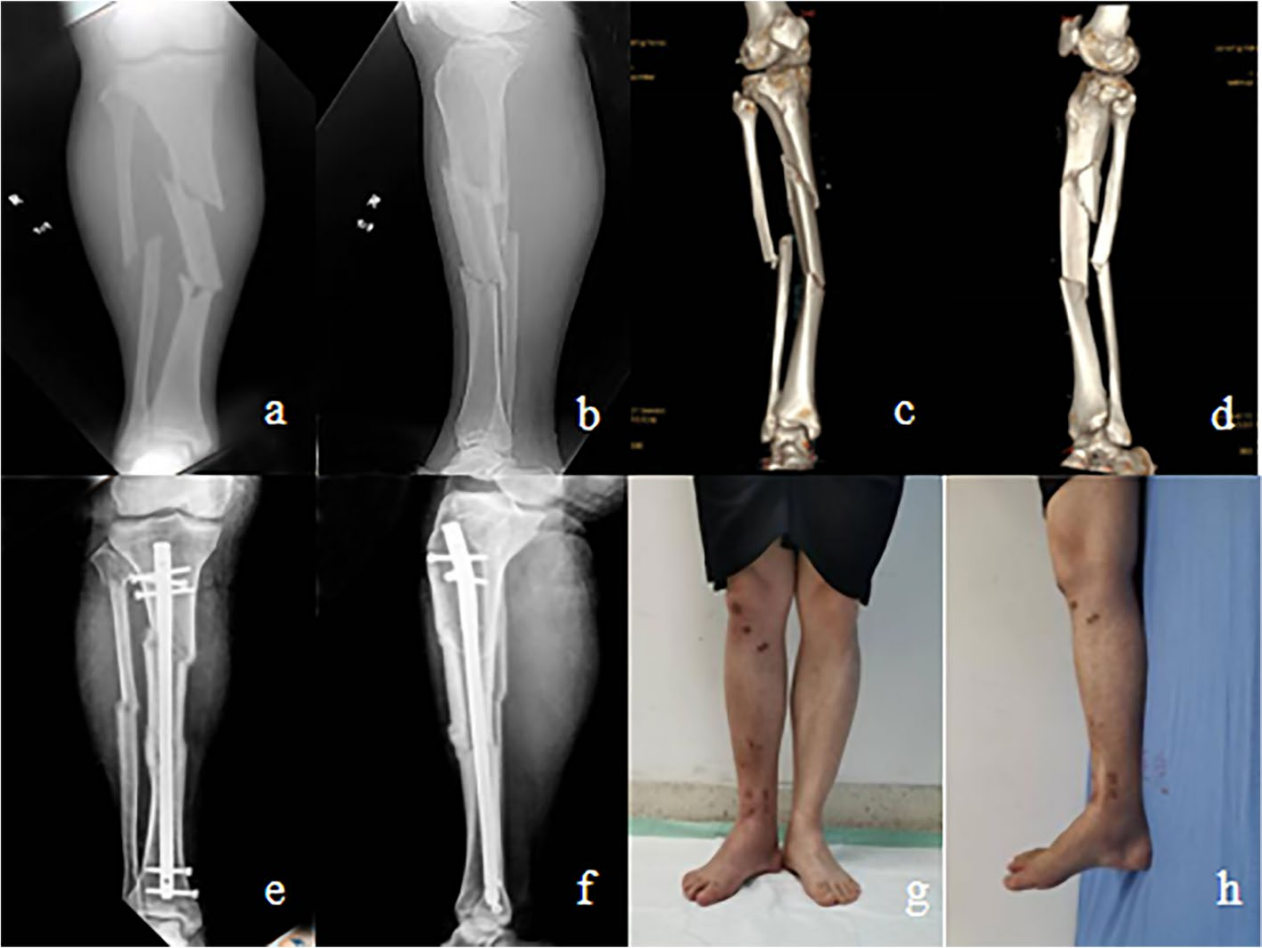
Images of treating a 48-year-old female with STFs due to traffic accident by using IMN. **a**, **b** X-ray images of injured STFs preoperatively. **c**, **d** Computed tomography reconstruction images of injured STFs preoperatively. **e**, **f** Images show good fracture healing 6 months postoperatively. **g**, **h** Clinical follow-up appearance images obtained 1 month after IMN removal

One typical STFs in IMN treatment is shown in Fig. 3.

## Discussion

STFs are caused by high-energy injuries, usually accompanied by severe peripheral soft tissue damage, and the incidence of open fractures is relatively high. Inherently, STFs have a higher risk of complications such as delayed union, malunion, nonunion, infection, and compartment syndrome [14]. At present, the treatment of STFs remains a major challenge for Orthopaedic physicians. Surgical treatment of STFs is the main treatment method [10, 15, 16]. The more commonly used fixation methods are IMN and HEF, both of which can be minimally invasive and have less interference with soft tissue. However, there is still controversy about which surgery is better for fracture reduction and has fewer postoperative complications in treating STFs [2, 15]. The results of this study indicate that the use of either HEF or IMN for STFs can achieve good therapeutic effects.

The proximal and distal medullary cavities of the tibia are relatively wide, and when intramedullary nails are inserted, their stability in tibial metaphyseal fractures decreases. It is often necessary to add blocking screws to increase stability. The surgical operation is complex, and repeated reduction also exacerbates damage to the blood supply at the fracture site. Therefore, based on the situation of both groups of patients, HEF has the following advantages: (1) HEF can use complete closed reduction with computer assistance, without the need to open and expose the fracture site, with minimal damage to the surrounding blood supply and only minimally invasive percutaneous insertion of fixation wires, and therefore causes minimal trauma. In this study, the HEF group achieved closed reduction of fractures without any cases of cutting the fracture site, protecting the blood supply around the fracture site and providing a good biological environment for fracture healing [12, 13]. (2) HEF can achieve stable fixation of the entire segment of the tibia, and the fracture site can be fixed through multi-plane penetrating fixation wires. The HEF has good stability and allows patients to bear weight on the ground early [10]. In this study, the HEF group had an earlier complete weight-bearing time and shorter bone union time than the IMN group. (3) The HEF group significantly improved the accuracy of fracture reduction [9], and the residual fracture displacement can be adjusted by measuring the AP and lateral radiographs with the aid of computer-based software postoperatively [12]. Effective fixation of the proximal 1/3 and distal 1/3 of the tibia is a difficult point in the treatment of segmental fractures, and the incidence of malunion of fractures in this area treated with intramedullary nails is as high as 84% [10]. In this study, the HEF group had better mMPTA, mPPTA, mLDTA, and mADTA than the IMN group, with better alignment. However, some problems were encountered using HEF to treat STFs. The study suggested that the ROM of dorsal flexion of the ankle joint in the IMN group was better than that in the HEF group, which may be due to the impact of olive wires on the tendon. Therefore, when threading wires around the ankle joint, it is necessary to bend or extend the ankle joint to maintain the maximum ROM.

STFs are usually caused by high-energy injury and are accompanied by different levels of soft tissue damage. The use of olive wires in the HEF group to fix the tibia can reduce both soft tissue damage and the incidence rate of complications such as intramedullary infection [9]. HEF can be applied to patients when intramedullary nails are not applicable, due to injuries at the insertion point and locking site, or when the tibial medullary cavity is too thin or too thick.

We understand that when using HEF to treat STFs, the following aspects should be noted: (1) The configuration of the HEF needs to be determined based on the position and quantity of the patient's fracture line, and there is no fixed configuration. In principle, each bone segment should be fixed with 1–2 rings. The metal ring should be fixed at both ends of each bone segment (distance principle) [17]. (2) Each ring has at least 3 fixed components, which can be reinforced with 2 olive wires and 1 HA-coated half wire. The olive wire near the closest end of the knee joint should be 1 cm below the joint surface of the tibial plateau, and a 2/3 ring should be used to prevent knee flexion from being restricted. For patients with osteoporosis, generally near the ankle or knee joint, three olive wires and 1–2 HA-coated half wires should be used for fixation. (3) Each bone segment is fixed with wires separately, making the metal ring as perpendicular to the bone segment as possible. Six quick universal adjustment rods are used to connect every two sets of rings, placed in a sliding state. The operator manually closed the metal ring at both ends of the fracture line for initial reduction, and residual deformities were reduced by measuring various parameters and inputting a computer software program. After satisfactory fracture reduction, quickly lock the nut on each quick universal adjustment rod [10, 18]. (4) When threading the wires, attention should be given to avoid important nerves and blood vessels, and the wires should be cooled with normal saline. Pin tract infection is a common complication in the application of HEF [19], which may be related to the high temperature generated during wire insertion, high skin tension, and inadequate wire care [20]. There was no deep wound infection in the HEF group, and 7 patients experienced superficial wire infection after the operation. The infection was controlled after pin-site dressing and oral antibiotics.

The present study has several limitations. First, this study is a retrospective comparative study, and there is randomness between the two groups; therefore, selection bias is inevitable. Second, the sample size of this study was small, and the number of included cases was limited, which may have led to deviations in the statistical analysis results. Therefore, it is necessary to expand the sample size to enrich this study and even multicenter studies to obtain more accurate conclusions.

## Conclusion

In terms of final clinical outcomes, the use of either HEF or IMN for STFs can achieve good therapeutic effects. While HEF is superior to IMN in terms of completely closed reduction, early full weight bearing, early bone union and alignment. Nevertheless, HEF has a greater impact on the ROM of dorsal flexion of the ankle joint, and much more care and adjustment are needed for the patients than IMN.

## Data Availability

A reasonable request for the datasets used in the current study can be made to the corresponding author.

## Abbreviations

STFs: Segmental tibial fractures
HEF: Hexaxial external fixator
IMN: Intramedullary nail
AO/OTA: Arbeitsgemeinschaftfür Osteosythese/Orthopaedic Trauma Association
mMPTA: Mechanical medial proximal tibial angle
mPPTA: Mechanical posterior proximal tibial angle
mLDTA: Mechanical lateral distal tibial angle
mADTA: Mechanical anterior distal tibial angle
HSS: Hospital for special surgery
AOFAS: American Orthopaedic Foot & Ankle Society
ROM: Range of motion
HA: Hydroxyapatite
AP: Anteroposterior
VSD: Vacuum sealing drainage
